# Short-term restoration effects of ecological projects detected using the turning point method in the Three River Headwater Region, China

**DOI:** 10.3389/fpls.2023.1239417

**Published:** 2023-10-12

**Authors:** Yuzhe Li, Yue Zhan

**Affiliations:** ^1^ Key Laboratory of Land Surface Pattern and Simulation, Institute of Geographical Sciences and Natural Resources Research, Chinese Academy of Sciences, Beijing, China; ^2^ College of Resources and Environment, University of Chinese Academy of Sciences, Beijing, China

**Keywords:** alpine grasslands restoration, ecological conservation and restoration projects, Mann-Kendall test, short-term effect, turning point detection, Three River Headwater Region

## Abstract

The Three River Headwater Region (TRHR) is an important river source area providing important ecological functions. Decades ago, climate change and human activities severely degraded the ecosystem in the TRHR. To restore vegetation, a series of ecological projects have been implemented since 1989. Using net primary productivity (NPP) data from 1988 to 2012, a sequential Mann–Kendall trend test (SQ-MK) method was applied to identify the turning point of vegetation NPP. This approach was able to represent the critical response time of the vegetation to important disturbances. A 3-year time window was set after the implementation of one ecological project to detect and analyze its short-term effects. The ecological projects included the Yangtze River Basin Shelterbelt System Construction Project (YRCP), the TRHR Nature Reserve Construction Project (TNR), the Returning Grazing Land to Grassland Project (RGLGP), and the first phase of the Ecological Conservation and Restoration Project of the TRHR (ECRP). Our results showed that the vegetation in the TRHR responded positively to restoration: 89% of pixels showed an increasing trend and 54% of pixels underwent an abrupt change. The accelerated growth type accounted for the highest proportion among all types of detected turning points. In the ECRP’s window, the positive turns rose rapidly, from 41% in 2005 to 86% in 2008, and it showed the most balanced restoration effects across grasslands. The alpine meadow and montane meadow restoration was largely influenced by the ECRP and the RGLGP (both >40%). The alpine steppe restoration was mainly attributed to the ECRP (68%). On the county scale, the positive turns in Yushu at the source of the Yangtze River mainly benefited from the RGLGP (56%), while the positive turns in Maduo at the source of the Yellow River benefited from the ECRP (77%). Nangqian, Tanggula and Zaduo County were still in need of intervention for restoration (< 3%). The results of the study can enhance our understanding of the spatio–temporal distribution of the short-term ecological benefits of different ecological projects, thus provide a scientific and timely reference for future planning and adjustment of the conservation and restoration projects.

## 1 Introduction

The Three River Headwater Region (TRHR), located in the hinterland of the Qinghai–Tibetan Plateau, is known as the “Water Tower of China” because of its crucial ecological functions and vital role in providing various ecosystem services for the whole country (Cao et al., 2020). Grassland is the dominant ecosystem type in the TRHR, and it is highly susceptible to the impacts of climate change and human disturbance (Liu et al., 2014; Zhai et al., 2020). Since the 1970s, the TRHR has experienced continuous vegetation degradation, resulting in biodiversity loss, soil erosion, wetland decline and other ecological problems(Liu et al., 2008a; Zhang et al., 2012). To protect and restore the fragile ecosystem, the Chinese government has launched a series of ecological projects. These projects aim to restore the degraded ecological environment through measures such as grazing prohibition, ecological migration, soil improvement and conservation. The projects include: the Yangtze River Basin Shelterbelt System Construction Project (YRCP) in 1989, the TRHR Nature Reserve Construction Project (TNR) in 2000, the Returning Grazing Land to Grassland Project (RGLGP) in 2003, and the first phase of the Ecological Conservation and Restoration Project of the TRHR (ECRP) in 2005 (Shi et al., 2011; Shao et al., 2013; Shao et al., 2016b; Shao et al., 2022).

To quantify the ecological benefits of ecological projects, and to understand the process and regional suitability of restoration measures, a large number of assessments and studies have been carried out in China and elsewhere. In 2002, the US Department of Agriculture launched the Conservation Effects Assessment Project, which assessed the impacts of four ecological conservation programs, such as the Wetlands Reserve Program, by comparing the project areas with the adjacent non-project areas, examining the changes before and after the project implementation, and contrasting the situations with and without the project interventions (Duriancik et al., 2008; Euliss et al., 2011). The Center for International Forestry Research used the Before–After–Control–Impact method to compare and assess the implementation effects of forest conservation projects REDD+ in 15 countries, and evaluated the role and contribution of the projects to carbon sequestration (Börner et al., 2016). The European Union implemented the Desertification Mitigation Assessment Project, which used a participatory evaluation to grade the effectiveness of specific measures for sand prevention and control in 12 countries (Rojo et al., 2012). China has carried out comprehensive benefit assessments of major projects such as the Three-North Shelterbelt System and the Grain for Green Project, and issued a series of National Reports on Ecological Benefit Monitoring of the Grain for Green Project (Administration, 2014) as well as evaluating the ecological effectiveness of ecological engineering in the Loess Plateau (Liu et al., 2017). In studies exploring the impacts of ecological engineering, vegetation characteristics (such as the leaf area index (Tong et al., 2018), vegetation productivity (Yang et al., 2014; Niu et al., 2019), vegetation coverage (Cai et al., 2022), and the vegetation index (Shen et al., 2018; Xu et al., 2020; Zhang et al., 2020)) are often used as the core ecological indicators for assessing the benefits of ecological projects. These indicators not only reflect the changes in vegetation, but also related closely to changes in ecosystem composition, structure and function (LaPaix et al., 2009; Terwayet Bayouli et al., 2021), as well as the environmental pressures that are associated with climate, hydrology and human activities (Ge et al., 2021). However, the restoration of vegetation is gradual and slow, thus it is necessary to identify and evaluate the long-term effects of a project by using long-term data.

Relying solely on long-term effect analysis not only leads to delayed evaluation of the outcomes of ecological projects, but also prevents timely and effective comparison and adjustment. At present, there are deficiencies in regional suitability assessment (Peng et al., 2016) and especially in the timely monitoring of measures (Yin et al., 2009; Liu et al., 2015) in ecological project assessment work. The impact and benefit assessment of ecological projects urgently needs more timely assessment methods. Large-scale analyses of remote sensing data that capture the short-term ecological effects of natural or human disturbances can be applied to monitor the changes in ecosystem conditions after sudden events. For example, Guzmán et al. monitored the short-term impact of oil spills on common shallow-water tidal reef coral (Guzmán et al., 1991). Lin et al. monitored and assessed vegetation recovery within two years of earthquake landslides (Lin et al., 2005). Han monitored vegetation dynamics in areas with different fire severity during the year after fire (Han et al., 2021). These studies all focused on detecting and identifying the rapid response of regional flora and fauna communities to habitat changes. In this study, the detected abrupt change of vegetation productivity was used as an indicator to achieve rapid detection of vegetation response after the implementation of ecological projects. The results obtained are the short-term effects of ecological restoration. We used the sequential Mann–Kendall trend test (SQ-MK) to identify significant turning points in NPP data series to examine vegetation fluctuations in a short period of time. The SQ-MK test is used to detect monotonic trends and turning points in time series data (Sneyers, 1991). It has no assumption for data distribution, high computational efficiency, easy implementation and the ability to identify the change of direction (Wei, 2007). Xulu et al. detected vegetation change using the SQ-MK test in South Africa (Xulu et al., 2021). Tong et al. quantified the effectiveness of ecological restoration projects in the karst regions of Southwest China (Tong et al., 2017). This detection method has been proven to rapidly identify the area and response time of vegetation to abrupt climate change or effective ecological projects. However, the applicability of this method to the high-altitude alpine grassland region, where field investigation and monitoring is more difficult, has not been reported yet. Based on Liu et al.’s findings, NPP is more sensitive to grazing intensity than NDVI in the Qinghai-Tibet Plateau (Liu et al., 2021). Therefore, we used NPP as the primary indicator of vegetation, as it represents both the net carbon sequestration by plants and the livestock production that we are interested in.

To assess the effectiveness and efficiency of the TNR, RGLGP and ECRP ecological projects in the TRHR, this study used NPP data from 1988 to 2012. The SQ-MK test and cluster analysis were applied to detect the trend and turning points in NPP to determine their occurrence and nature. The objectives of this study were to 1) analyze the spatial and temporal variability of the short-term effects and the process of different ecological projects; 2) assess the regional short-term effects of ecological projects after implementation; and 3) explore the reasons for the differences in the short-term effects of different ecological projects and the implications for subsequent restoration. The results of the study can provide a reference for assessing the short-term effects of major ecological projects.

## 2 Materials and methods

### 2.1 Study sites

**Figure 1:**
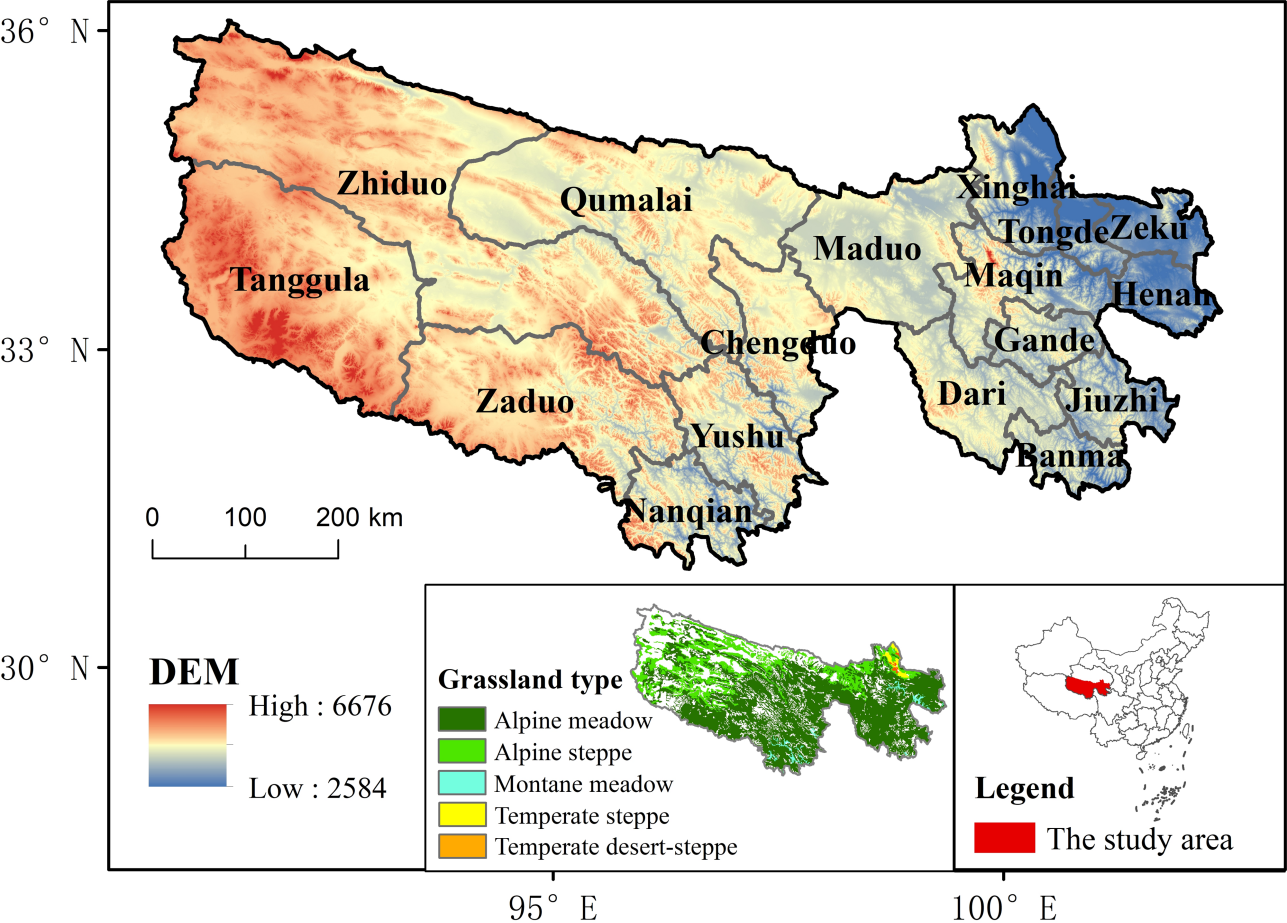
Grassland types and location of the Three River Headwater Region.

The TRHR is found in southern Qinghai Province, China, a mountainous area of 35.66×104 km^2^ between 31°39’–36°12’ N and 89°45’–102°23’ E. The average altitude is 4588 m and the climate is cold and dry with strong radiation. The multi-year average temperature is −5.38°C to 4.14°C and the precipitation is 262.2 mm to 772.8 mm (McGregor, 2016). The TRHR is in the source area of the Yangtze river, Yellow river, and Lancang river. It not only the largest nature reserve in China, but also a key area for global ecological functions (**Figure 1**). There are ten vegetation types distributed in the TRHR and grassland is the main ecosystem component (Department of Animal Husbandry and Veterinary, 1996). Grassland covers 26.27 million ha, accounting for 73.65% of the total area. In this study, the five major grassland types were selected. The area of alpine meadow was the largest, accounting for 74.79%, followed by alpine steppe, montane meadow, temperate steppe, temperate desert–steppe, accounting for 22.44%, 1.66%, 0.88%, and 0.24%, respectively.

### 2.2 NPP data

In this study, a 1988–2012 1000m resolution raster dataset of net primary productivity of vegetation in the TRHR was used. The data were obtained from the National Platform for Basic Conditions of Science and Technology–National Earth System Science Data Sharing Service Platform (http://www.geodata.cn). The Global Production Efficiency Model (GLO-PEM) (Prince, 1991; Prince and Goward, 1995; Goetz et al., 2000) is a novel model that estimates both net and gross primary production of terrestrial ecosystems at the global scale by using the production efficiency concept. The NPP dataset was calculated using absorbed photosynthetically active radiation (APAR) with the environmental factors that affect the efficiency of APAR conversion in the GLO-PEM model (Pinker et al., 2010; Wang et al., 2017). This data is reliable for ecological geography studies. It reflects the productivity of plant communities and the quality of terrestrial ecosystems (Shao et al., 2016b; Shao et al., 2016c; Shao et al., 2017; Liu et al., 2018).

### 2.3 SQ-MK test

In this study the SQ-MK test (Sneyers, 1991) was used to detect approximate trend turning points. It is based on the Mann-Kendall test, which is widely used to analyze the monotonic trends in hydrological and climatological data. Furthermore, it can identify the approximate turning points of a trend by applying the Mann-Kendall test to sequential segments of the data. The advantage of the SQ-MK test is that it has no assumption for data distribution, high computational efficiency, easy implementation and the ability to identify the change of direction (Wei, 2007). The formula for the SQ-MK is as follows:


(1) $$t_{i}=\sum_{\text{j}=1}^{\text{i}}n_{i}$$

(2) $$\text{E}\left(t_{i}\right)=\frac{\text{i}\left(\text{i}-1\right)}{4}$$

(3) $$\text{Var}\left(t_{i}\right)=\frac{\text{i}\left(\text{i}-1\right)\left(2\text{i}-5\right)}{72}$$

(4) $$\text{U}\left(t_{i}\right)=\frac{t_{i}-\text{E}(t_{i})}{\sqrt{\text{Var}(t_{i})}}$$

The values of y_i_, (i = 1, 2, 3,…, n) are compared with y_j_, (j = 1, 2, 3,…, j−1), by using the ranked values of y_i_ from a given time series (x_1_, x_2_, x_3_,…, x_n_). In each comparison, the number of cases where y_i_ > y_j_ is counted and assigned to n_i_. E(t_i_) and Var(t_i_) is the mean and variance of the statistic t_i_ respectively. U(t_i_) is the standardized values of the statistic. Then, this method produces a forward trend (UF(t_i_)) and a backward trend (UB(t_i_)) of the time series calculated at the same time, but the UB(t_i_) values are computed by starting from the end of the time series. UF(t_i_) is an indicator of a positive NPP trend, while a negative UF indicates a declining NPP trend. The region where the indicators cross and diverge beyond the specified threshold ( ± 1.96 in this study) is regarded as a statistically significant trend, with the point of intersection indicating the start of a turning point in the trend.

**Figure 2:**
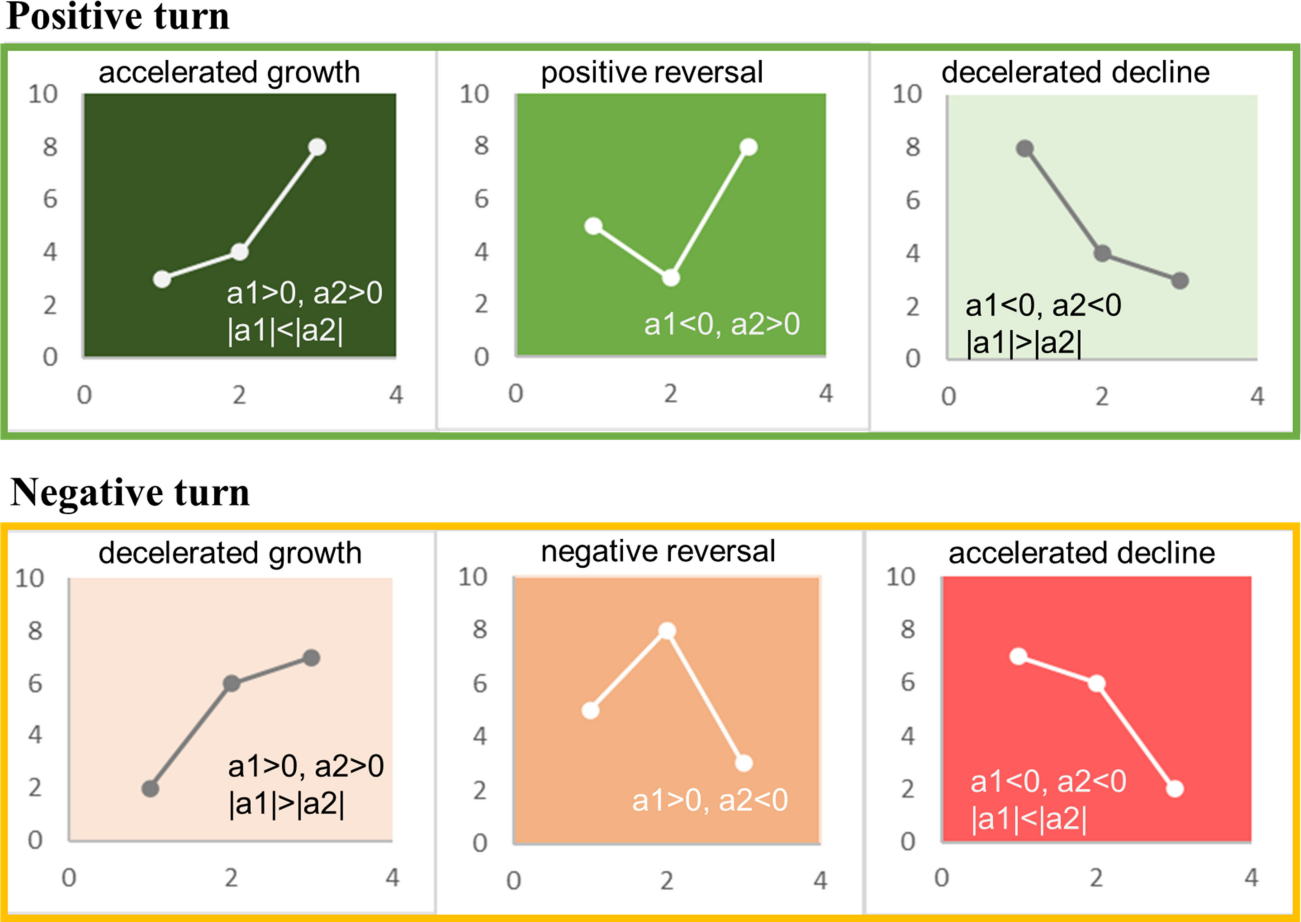
Types of turning points.

To detect the type of turning point, we employed a segmented linear fitting approach, which involved comparing the linear fitting slopes before and after the turning point for each pixel. Assuming that the slope of the linear fitting before the turning point was a1 and the slope after the inflection point was a2, we defined the following criteria (**Figure 2**): if a1 > 0 and a2 > 0 and |a1|< |a2|, the type was “accelerated growth”, and if |a1| > |a2|, the type was “decelerated growth”. If a1< 0 and a2 > 0, the type was “positive reversal”. If a1< 0 and a2< 0 and |a1|< |a2|, the type was “accelerated decline”, and if |a1| > |a2|, the type was “decelerated decline”. If a1 > 0 and a2< 0, the type was “negative reversal”. Among the six types, accelerated growth, positive reversal and decelerated decline were defined as a positive turn, and the others were defined as a negative turn.

### 2.4 Regional short-term effects analysis

Based on positive turn, two indicators were used to compare and analyze the short-term effects of different ecological projects on regions (grassland types/counties). PT_A_ was the proportion of positive turn pixels to the total number of pixels in a region, reflecting the overall level of grassland restoration in that region. PT_P_ was the proportion of positive turn pixels from one project’s window (the 3-year time window) to the total positive turn pixels in three ecological projects’ windows (TNR, RGLGP, ECRP), reflecting the relative contribution of a specific project to grassland restoration in that region. The formulas are as follows:


(5) $$PT_{A}=\;{\text{N}}_{\text{P}\left(\text{i}\right)}/N_{A}$$

(6) $$PT_{P}=\;{\text{N}}_{\text{P}\left(\text{i}\right)}/\bar{N_{P}}$$

where N_P(i)_ represents the number of positive turn pixels in the region with a certain project’s window. N_A_ represents the total number of pixels in the region. $\bar{{\text{N}}_{\text{P}}}$ is the sum of positive turn pixels in the region within the three projects’ windows.

### 2.5 Cluster distribution analysis

Spatial autocorrelation on the basis of feature locations and attribute values was measured using the Global Moran’s I statistic. If the p-value is very small and the z-score is either very high or very low, then the pattern exhibited is not consistent with the theoretical random pattern represented by the null hypothesis, indicating a tendency toward clustering (Esri, 2021b).

The Optimized Hot Spot Analysis tool can identify statistically significant spatial clusters of high values (hot spots) and low values (cold spots). In this study we applied this method to examine the spatial variation of the timing of the turning points (Esri, 2021a).

## 3 Results

### 3.1 Spatial patterns of turning points in the TRHR

**Figure 3:**
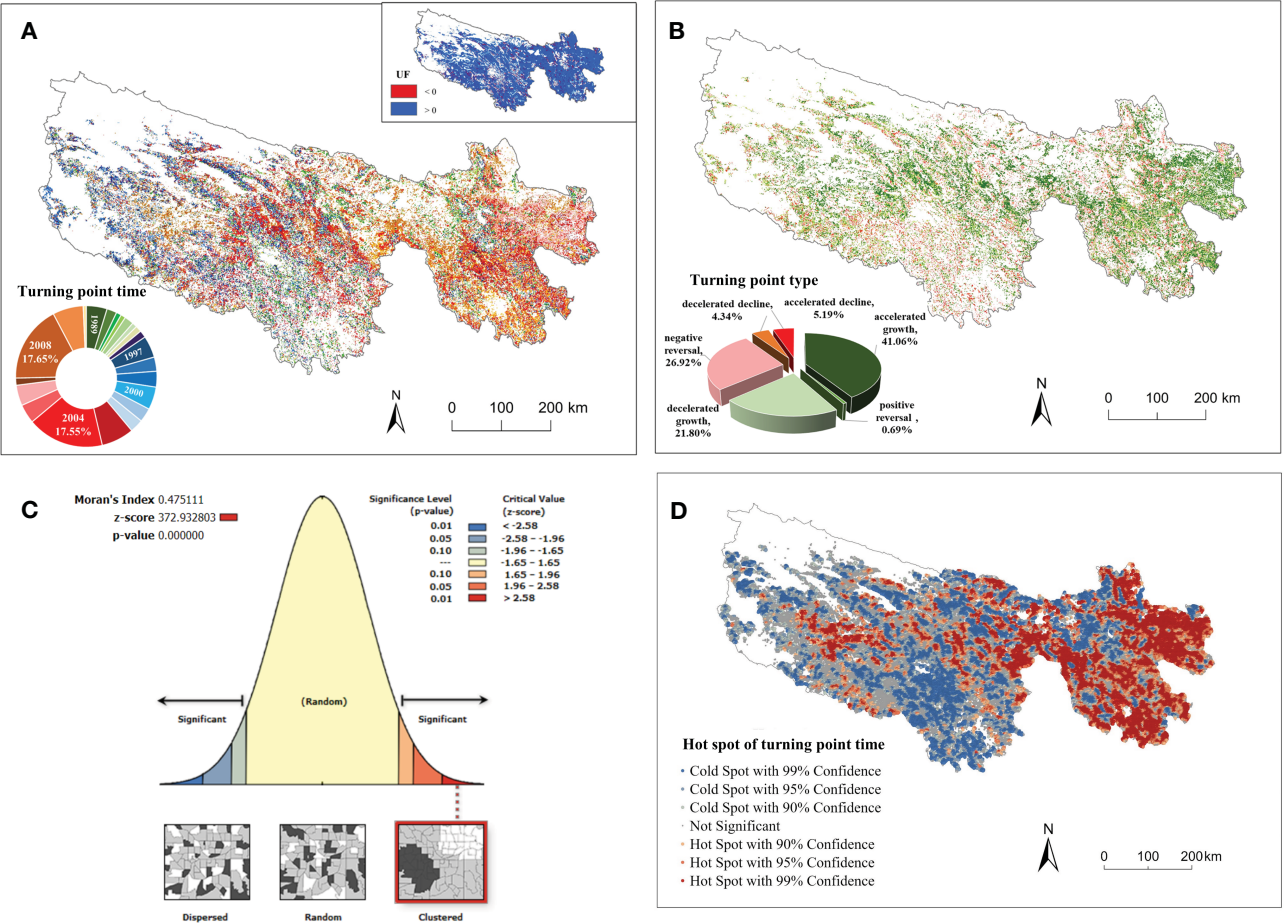
The spatial patterns of turning point time **(A)** and turning point types **(B)**; Global Moran’s I result **(C)** and the hot spot results **(D)** of turning points in the TRHR.

The results of the SQ-MK test pixel by pixel are shown in **Figure 3A**. The vegetation in the TRHR exhibited an increasing trend, with positive trend (UF>0) in 89.07% of the pixels and turning points in 54.47%. In general, 13.96% of the turning points occurred in 1989–1995, which were concentrated in the northern and southern parts of the TRHR. A further 24.95% occurred in 1996–2002, which were more prevalent in the western part; while 34.10% occurred in 2003–2007, which were mainly located in the eastern and central parts. Approximately 27% occurred in 2008–2010, which were situated in the southeast and extended to the central part. It can be observed that the number of turning points increased after 2000. The largest proportion of turning points occurred in 2008, accounting for 17.65% of the total, followed by 2004 with 17.55%.

From the perspective of turning point types (**Figure 3B**), the most dominant type was accelerated growth (41.06%), which spanned from the east to the west. Negative reversal, accounting for 26.92%, occurred primarily in the southern and northern parts. The other types of turning points were decelerated growth (21.80%), accelerated decline (5.19%), decelerated decline (4.34%) and positive reversal (0.69%).

The results of Global Moran’s I (**Figure 3C**) statistic identified a significant positive spatial autocorrelation pattern with a large z-value and a small p-value, indicating that similar turning values tended to be clustered. According to the results of the Optimized Hot Spot Analysis (**Figure 3D**), the eastern part was a significant hot spot area and there were local hot spot clusters in the western part. The southwestern part was a significant cold spot area. This implied that the turning point time in the TRHR exhibited a delay from south to north and from west to east.

### 3.2 Turning points of different grassland types in the TRHR

**Figure 4:**
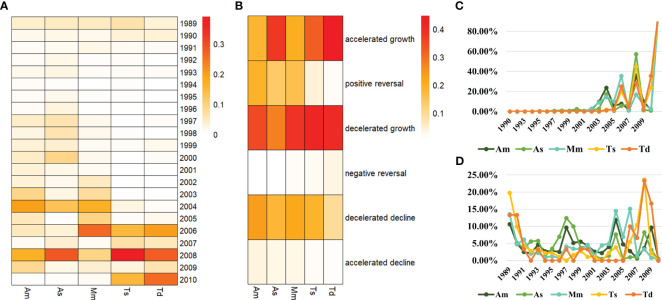
Turning point time **(A)**, turning point type **(B)**, the percentage of positive turns **(C)** and the percentage of negative turns **(D)** of different grassland types in the TRHR. Am, alpine meadow; As, alpine steppe; Mm, montane meadow; Ts, temperate steppe; Td, temperate desert-steppe.

The turning point time of different grassland types was analyzed (**Figure 4A**). The most turning points for alpine meadow occurred in 2004 and 2008, accounting for 19.75% and 16.89%, respectively. For alpine steppe, the most turning points occurred in 2008 and 2004, accounting for 28.72% and 12.85%, respectively. For montane meadow, the most turning points were recorded in 2006 and 2004, accounting for 26.70% and 16.73%, respectively, while the turning points in other years were all less than 10%. The most turning points for temperate steppe occurred in 2008, 2010 and 2006, accounting for 38.90%, 15.10% and 14.92%, respectively and those for temperate desert–steppe also occurred in 2008, 2010 and 2006, accounting for 27.97%, 26.27% and 20.34%, respectively. Generally, alpine meadow, montane meadow and alpine steppe had earlier turning point time, while temperate steppe and temperate desert–steppe had later turning point time.

Comparing the turning point types (**Figure 4B**), it was observed that the majority of pixels in alpine meadow were the decelerated growth type, reaching 35.29%; followed by decelerated decline with 23.36%, positive reversal with 19.15% and accelerated growth type with 17.97%, while the other types were all less than 5%. The majority of pixels in alpine steppe were the accelerated growth type, accounting for 37.76%, while decelerated growth accounted for 27.14%, decelerated decline accounted for 17.59%, positive reversal accounted for 13.71%, and the other types were all less than 5%. The majority of pixels in montane meadow were the decelerated growth type, reaching 38.59%; followed by decelerated decline with 21.14%, accelerated growth type with 19.94% and positive reversal with 15.83%, while the other types were all less than 5%. The temperate steppe also had a majority of pixels of the decelerated growth type, reaching 41.27%; followed by accelerated growth with 32.29% and decelerated decline with 18.70%, while the other types were all less than 5%. The majority of pixels in the temperate desert–steppe was the accelerated growth type, up to 44.56%, while decelerated growth type made up 40.41%, and the other types were all less than 10%.

The proportion of positive turns in different grassland types (**Figure 4C**) showed that there were obvious peaks in 2004, 2006 and 2008, and that the peak in 2008 was higher than that in 2004 and 2006. The grassland types that had positive turn peaks in 2004 (>10%) were: alpine meadow, alpine steppe and montane meadow. The grassland types that had positive turn peaks in 2006 (>20%) were: montane meadow, temperate desert–steppe and temperate steppe. The grassland types that had positive turn peaks in 2008 (>30%) were: alpine steppe, temperate steppe and alpine meadow. The proportion of negative turns for different grassland types (**Figure 4D**) showed that there were significant peaks in 1989, 1997, 2004, 2006 and 2008, but the proportions were all less than 25%. In 1989, the negative turns of all grassland types were greater than 10%, and in 1997, the negative turns of alpine steppe and alpine meadow were both greater than 9.00%. In 2004, the grassland types with negative turn peaks (>10%) were: montane meadow and alpine meadow. In 2006, the grassland types with negative turn peaks (>10%) were: montane meadow and temperate desert–steppe. In 2008, the negative turns of temperate steppe and temperate desert–steppe were both greater than 20%.

### 3.3 Turning points of different counties in the TRHR

**Figure 5:**
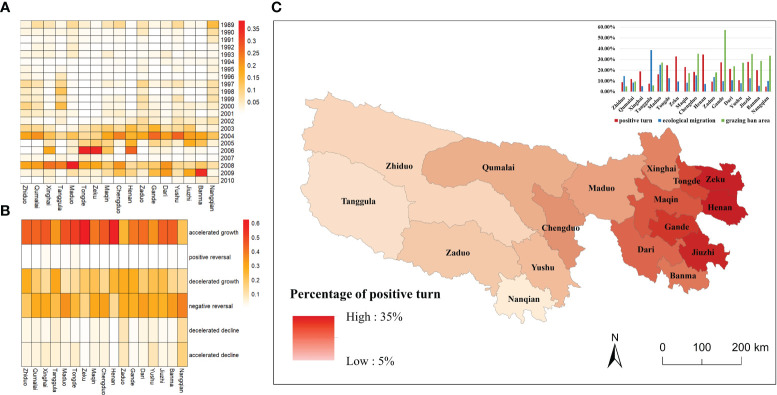
Turning point time **(A)** and turning point type **(B)** of different counties; the spatial pattern and percentage of positive turn (accelerated growth and positive reversal), ecological migration and grazing ban area **(C)** of different counties in the TRHR.

From the comparison of the turning points of each county, the years with more than 10% of the turning points were used to show the major turning point times for each county (**Figure 5A**). The major turning point times for both Zhiduo and Qumalai were in 2004 and 2008; Tanggula was 1998, 2000 and 2008; Xinghai and Maduo were 2004 and 2008; Tongde was 2006 and 2008; Zeku was 2005, 2006 and 2008; Maqin was 2003, 2004, 2006 and 2008, Chengduo was 2004, 2008 and 2009, Henan was 2003, 2004, 2005 and 2006, Zaduo was 2004 only, Gande was 2003, 2004, 2008 and 2009, Dari was 2004, 2008 and 2009, Yushu was 2004, Jiuzhi was 2003, 2004, 2005 and 2009, Banma was 2004, 2005, 2008 and 2009; and Nangqian was 1989 and 2004. In terms of turning point types (**Figure 5B**), only Zaduo had a majority of pixels with decelerated growth. The other counties all had a majority of pixels with accelerated growth, with the highest proportion in Zeku of 62.21%.

The spatial distribution and the proportion of positive turns in each county was analyzed (**Figure 5C**). The proportion of positive turns decreased from east to west. Henan County had the highest proportion, accounting for 34.74% of the total area, while Zeku, Jiuzhi and Gande all exceeded 25%. Zaduo, Zhiduo, Tanggula and Nangqian all had proportions less than 10%, among which Nangqian had the lowest proportion, only accounting for 4.71% of the total area. According to the collected data of ecological projects during 2003–2005, Tanggula, Maduo and Chengduo had more than 15% of ecological migration, while Gande, Chengduo and Jiuzhi had more than 35% of the grazing ban area.

### 3.4 Short-term effects of ecological projects

**Figure 6:**
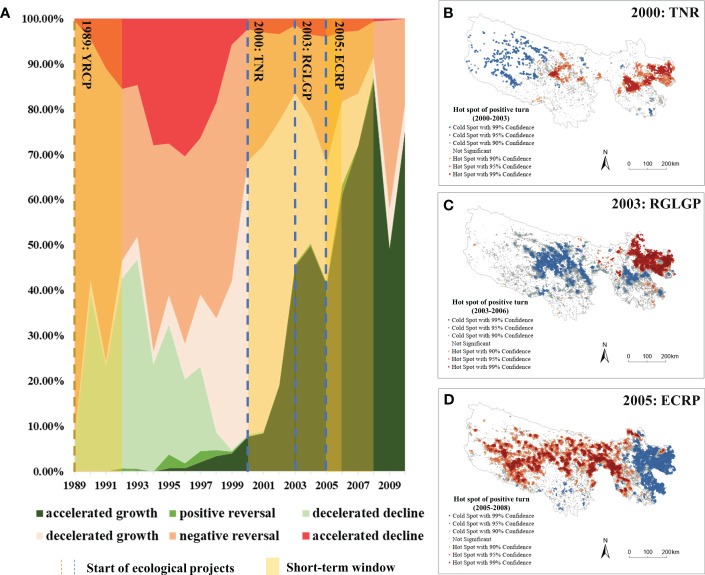
The percentage of turning point types in each turning point year **(A)**; hot spot results of positive turns during 2000–2003 (TNR) **(B)**, 2003–2006 (RGLGP) **(C)** and 2005–2008 (ECRP) **(D)** in the TRHR.

The turning point types corresponding to each turning point time were analyzed (**Figure 6A**), and the short-term effect of each major ecological project was identified by applying a 3-year time window. It can be seen that there was no improvement in vegetation in the YRCP’s window for shelterbelts in 1989, because grassland was the dominant vegetation type in the TRHR. Therefore, the following short-term effect analysis only considered the TNR, RGLGP and ECRP.

In the TNR’s window, the proportion of turning points with decelerated growth increased, but so did the accelerated growth, which rose rapidly from 2000 to 2003 and reached 45.39% in 2003. The implementation of the RGLGP in 2003 stabilized the proportion of accelerated growth at approximately 45.00% until 2006. The launch of ECRP in 2005 boosted the proportion of accelerated growth again, from 41.32% in 2005 to 86.22% in 2008. These results indicated a clear trend of rapid vegetation recovery after each project. Spatially, the western part of the region had earlier turning points than the eastern part in the TNR’s window (2000–2003) (**Figure 6B**) and the RGLGP’s window (2003–2006) (**Figure 6C**), while the opposite was true in the ECRP’s window (2005–2008) (**Figure 6D**).

### 3.5 Regional differences in short-term effects

**Figure 7:**
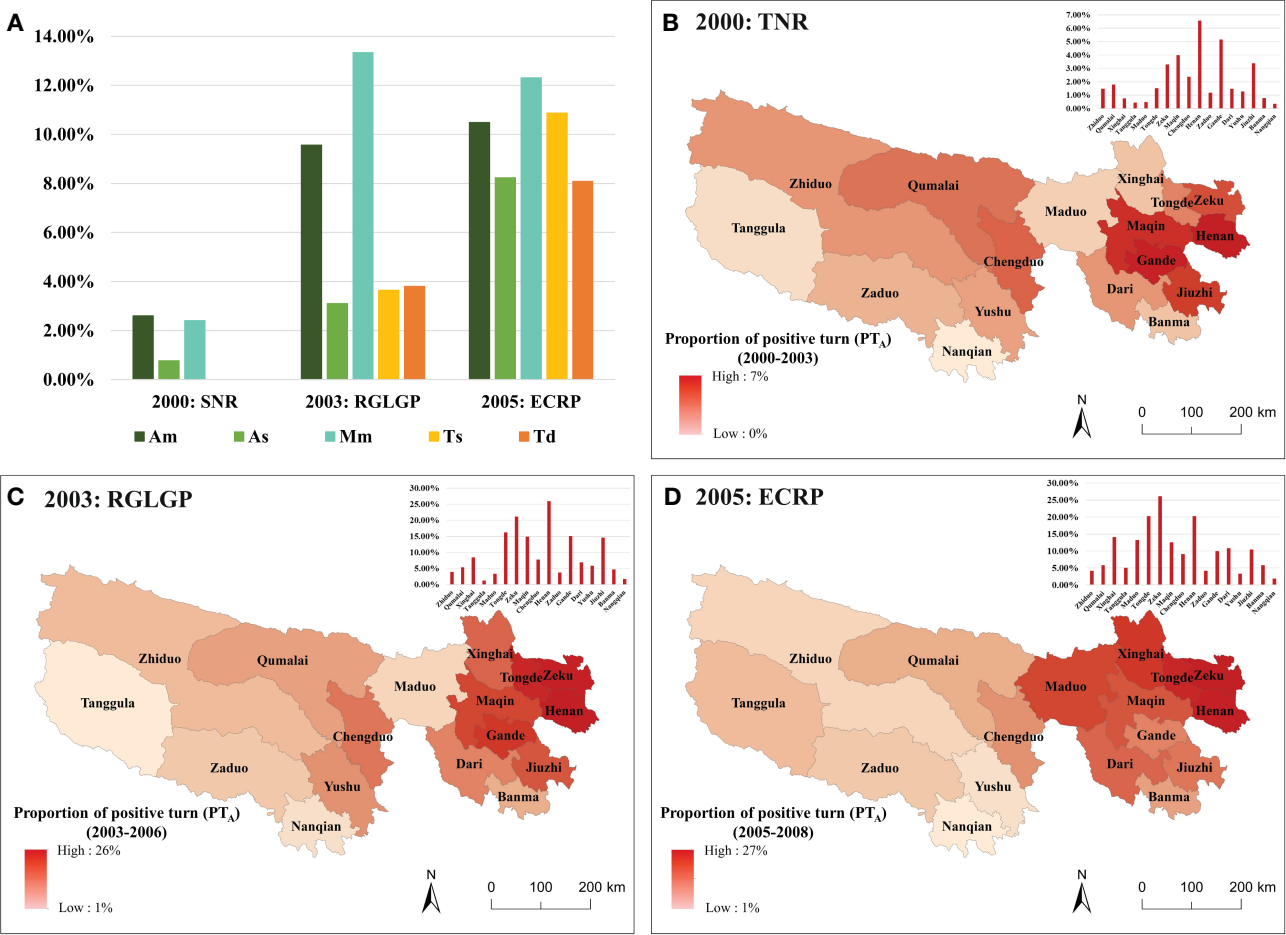
The PT_A_ of different grasslands **(A)**; the spatial pattern of PTA during 2000–2003 (TNR) **(B)**, 2003–2006 (RGLGP) **(C)** and 2005–2008 (ECRP) **(D)** of different counties. Am, alpine meadow; As, alpine steppe; Mm, montane meadow; Ts, temperate steppe; Td, temperate desert–steppe.

To compare the short-term responses of different grassland types, we calculated the PT_A_ for each grassland type in the TNR’s, RGLGP’s and ECRP’s window (**Figure 7A**). In the TNR’s window, the PT_A_ of grasslands was not high, with most positive turn in alpine meadow and montane meadow. In the RGLGP’s window, montane meadow had the highest PT_A_, reaching 13.33%, followed by alpine meadow, reaching 9.56%. Other grassland types were approximately 3.5%. In the ECRP’s window, the PT_A_ for different grassland types was similar, with montane meadow still having the highest proportion, reaching 12.32%, followed by temperate steppe (10.88%), alpine meadow (10.50%), alpine steppe (8.23%) and temperate desert–steppe (8.10%). The county-level analysis of the short-term effects revealed that the PT_A_ varied across different regions in different windows. In the TNR’s window (**Figure 7B**), Henan (6.58%), Gande (5.15%), and Maqin (3.99%) were the three counties with the highest PT_A_. In the RGLGP’s window (**Figure 7C**), the counties with the highest PT_A_ were Henan (25.99%), Zeku (21.16%), and Tongde (16.30%), while in the ECRP’s window (**Figure 7D**), they were Zeku (26.08%), Henan (20.23%), and Tongde (20.20%).

**Figure 8:**
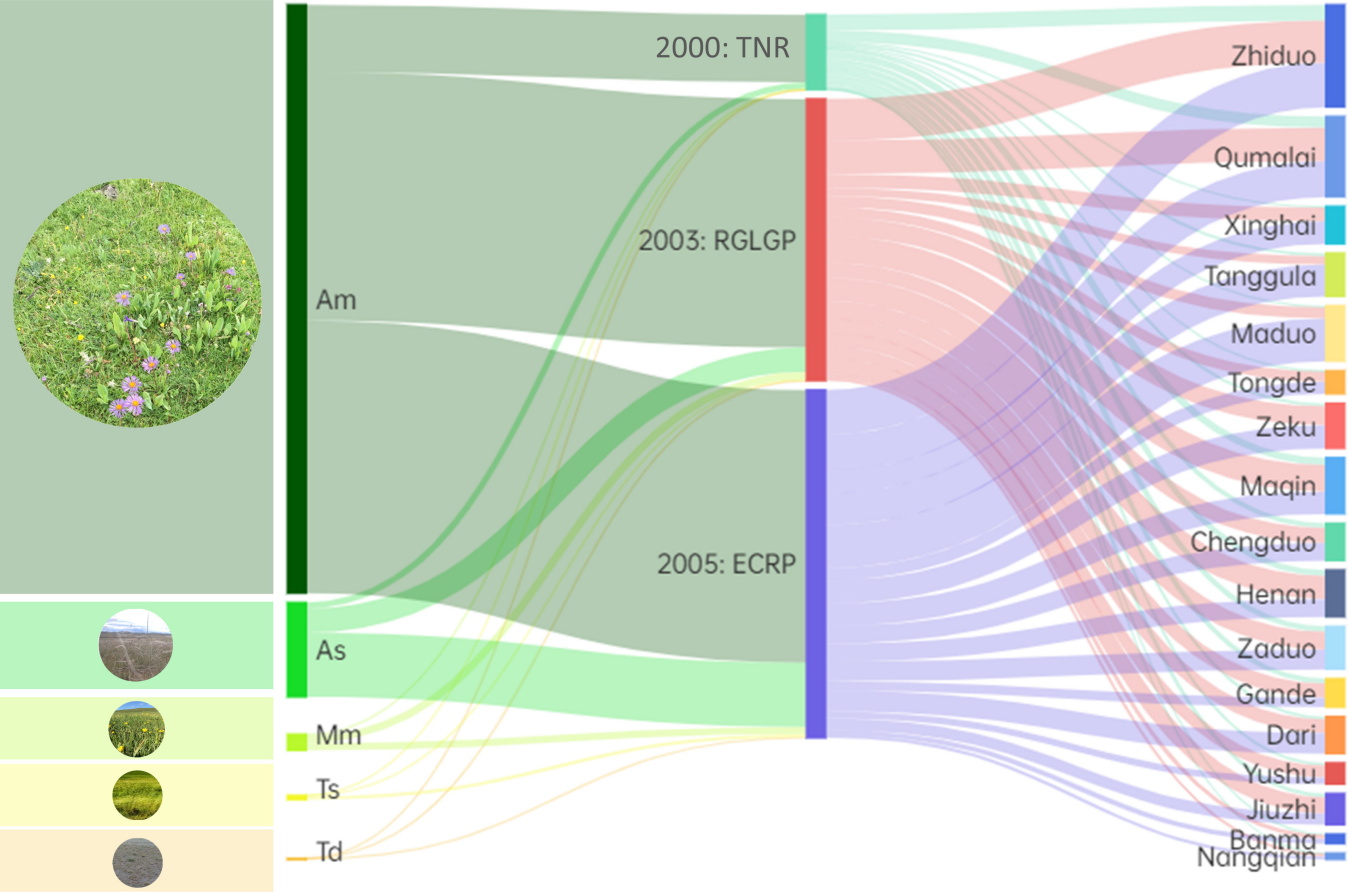
The source–destination diagrams of the positive turns from 2000–2003 (TNR), 2003–2006 (RGLGP) and 2005–2008 (ECRP) of different grasslands and different counties. Am, alpine meadow; As, alpine steppe; Mm, montane meadow; Ts, temperate steppe; Td, temperate desert–steppe.

An analysis of the source–destination diagrams shows the composition of the positive turns in the 3-year time window of the three projects (**Figure 8**). In terms of grassland types, the positive turn for montane meadow mainly came from the implementation of the RGLGP and ECRP, and the PT_P_ was 47.53% and 43.90%, respectively. The same was true for alpine meadow, accounting for 42.32% and 46.32%, respectively. For temperate steppe, temperate desert–steppe and alpine steppe, their positive turns mainly came from the implementation of the ECRP, accounting for 74.92%, 68.00% and 67.94%, respectively. At the county level, Yushu, Jiuzhi, Gande, Henan and Maqin were most affected by the implementation of the RGLGP, with a PT_P_ higher than 47%. Most other counties were mainly affected by the implementation of the ECRP, with a PT_P_ higher than 48%.

## 4 Discussion

### 4.1 Assessment of the short-term effects of restoration with turning point analysis

Combining the long-term NPP data from 1988 to 2012 and the SQ-MK test to obtain the turning point time and turning point type on a pixel-by-pixel basis, the results showed that the vegetation in the TRHR tended to have a positive response to ecological restoration during the past 25 years. Since 2000, a series of ecological projects for grassland restoration has been carried out in the TRHR. The study found that 72.56% of the pixels had a turning point in the TRHR after 2000 (**Figure 3A**), 73.55% of which were positive turn (**Figure 3B**). A study that compared remote sensing images in 2004 and 2012 confirmed that, the overall vegetation in the TRHR was in a stage where the degradation was limited to an initial stage, and some areas showed signs of improvement (Xu et al., 2017). We also found that the negative reversal rate was 26.92%, which means that there was a degradation in some areas. It is consistent with the findings of Shen et al., who compared the vegetation changes inside and outside the TRHR Reserve from 2005 to 2015. They found that the vegetation in some non-protected areas had become severely degraded (Shen et al., 2018).

Vegetation change is driven jointly by human activities and climate change, and the long-term trend in regional vegetation is influenced by the changing climate trend (Ge et al., 2021; Shi et al., 2021). To distinguish and identify the impact of the implementation of ecological projects on vegetation restoration more clearly, the study conducted a short-term analysis (the 3-year time window) of the four ecological projects implemented in the TRHR from 1988 to 2012 (**Figure 6A**). The study found that the YRCP implemented in 1989 with a long-term forest protection goal was the only project that did not significantly improve the vegetation in short term. This was because it was difficult to afforest the TRHR owing to its high altitude and its poor forestry basis (Zhang and Li, 2001). The TNR in 2000, the RGLGP in 2003 and the ECRP in 2005 did improve vegetation in a short period of time, but there were differences among them. The proportion of positive turns in the TNR’s window increased rapidly, from 7.71% in 2000 to 45.39% in 2003. By protecting and rebuilding the balance of the ecological environment, the TNR reversed the rapid decline in biodiversity and curbed the degradation of the ecological environment in the TRHR (Yin et al., 2001). The positive turns in the RGLGP’s window showed a slight but fluctuating increase in the short term with a mean rate of 49.41%. One study show that since the RGLGP was implemented in 2003, the grassland coverage in the TRHR increased by 15% to 20% (Wang and Rong, 2012). The proportion of positive turns in the ECRP’s window increased significantly, from 41.32% in 2005 to 86.22% in 2008. Relevant studies found that since the implementation of the ECRP in 2005, the percentage of grasslands with excellent, good, and medium grass conditions had all increased significantly (Wang et al., 2022), which is consistent with the short-term effects observed in this study. From the four cases cited above, it can be inferred that a short-term window is applicable to identify the short-term effects of ecological projects.

Previous studies have used the difference analysis method to compare the short-term effects of ecological projects in the TRHR. The differences in the three-year average NDVI of different projects were used to analyze the vegetation status (Zhai et al., 2020). This method could capture the extent of the vegetation change in a specific period, but it had limited ability to detect and identify the areas with less impact from the projects, because the short-term window resulted in only small changes to the NDVI value of the vegetation. In our study, the turning point detection method was more sensitive and able to effectively capture the short-term spatio–temporal changes from vegetation which was influenced by ecological projects.

### 4.2 Varied short-term effects of different ecological projects

The ecological projects implemented in the TRHR targeted different grassland types for restoration and improvement, and these grasslands varied significantly among the projects (**Figure 7A**). The TNR had an impact only on montane meadow, alpine meadow and alpine steppe in the short term (all<3%), possibly because the nature reserve was mainly established in the west of the TRHR to protect the threatened animal and plant resources as well as water resources(He, 2001). Therefore, only the three grasslands mentioned above, located in the core of nature reserve, recovered rapidly in the early stage (**Figure 1**). The RGLGP significantly recovered montane meadow (13.33%) and alpine meadow (9.56%) in the short term. A previous study indicated that the optimal period of grazing prohibition in alpine meadows should be 5–7 years according to the complexity of plant communities and changes in key species (Zhang et al., 2021). However, in this study, it was found that the NPP of meadow grasslands recovered significantly within 3 years, as detected by the turning point method. The reason could be attributed to the rapid response to composite interventions in the grasslands because the RGLGP incorporated other measures such as over-sowing and fertilization (Shao et al., 2016a). In the short term, the ECRP had obvious recovery effects on all the grassland types and higher proportions of positive turns were witnessed in alpine meadows (12.32%) and temperate steppe (10.88%). The measures implemented by the ECRP, such as ecological migration, ecological compensation, and grazing prohibition and enclosure, were focused on the densely populated areas in the eastern part of the TRHR (Shao et al., 2017; Su et al., 2021). The livestock pressure on grasslands in grazing areas was significantly reduced (Liu et al., 2013; Shao et al., 2017), enabling the high productivity grasslands in the eastern part to show a stronger recovery trend in the short term. In summary, ecological restoration plans should be designed and adjusted based on the specific characteristics and needs of different grassland types and regions, and suitable restoration actions and periods should be selected, to prevent over-interference or omission of certain areas.

The positive effects of vegetation restoration induced by the ecological projects had different temporal and spatial development processes in the TRHR (**Figure 5B**). After the implementation of the TNR and the RGLGP, the positive turns first occurred in the west (**Figures 6B, C**), while after the implementation of the ECRP, the positive turns first occurred in the east (**Figure 6D**). Most long-term studies were able to record vegetation restoration trends and long-term changes in related indicators, but failed to detect the timing and process of restoration effects in short term (Shao et al., 2016b). The detection of short-term effects obtained in this study contributed to an understanding of the implementation and effectiveness process of various ecological projects in this region. The start-up process of the restoration effects might differ owing to the variations in the restoration measures of each project, the distribution of vegetation types in each region, and the composition of dominant species of grassland types. These phenomena indicate that different vegetation types might need different time spans to respond to the restoration measures of the projects. In addition, previous studies indicated that the response times to the restoration measures vary depending on the life history strategies of species that experience environmental disturbance (Albaladejo-Robles et al., 2023): perennial plants tended to respond more slowly than annual and short-lived plants (Yachi and Loreau, 1999; Lan and Zhang, 2008; Ma et al., 2017), and weeds in different plant functional groups tended to respond faster than grasses (Li et al., 2021).

By comparing the effects of different projects, it can be seen that the TNR, which was a protective project, had a lower short-term effect than the RGLGP and ECRP, both restorative projects (**Figure 8**). Protective ecological projects were more in line with the law of natural succession, but they could not completely prevent the impact of external disturbances and pressures on the ecosystem, nor could they restore the ecological functions and services that had disappeared (Tao et al., 2022). In 2000, the grasslands of the TRHR had formed a degradation pattern(Liu et al., 2008b), and at this time, restorative ecological projects were needed to artificially intervene and rebuild the severely damaged ecosystem (Rohr et al., 2018). Some parts of the TRHR grasslands that had reversed the degradation pattern (Shao et al., 2017) should receive protection-oriented management and utilization in the later stage (Li et al., 2012). When designing and implementing future ecological projects, different evaluation criteria and methods should be applied according to ecological projects of different types, and then integrated with the regional context, to select the most suitable ecological measures.

### 4.3 Varied regional responses of short-term effects in different ecological projects

Since the implementation of the ecological projects, the proportions of detectable vegetation restoration were different in each county in the TRHR (**Figure 7**). Evaluating the differences in the generation process and comparing composition of the positive turns in each county (**Figure 8**) can help to identify effective restoration measures in the region to promote and improve the implementation efficiency of follow-up projects. In the TNR’s and RGLGP’s window, the short-term effects were more obvious in the eastern counties of the TRHR (**Figures 7B, C**), which was consistent with the results of long-term observations (Zhai et al., 2020). Further analysis found that the positive turns in eastern counties such as Henan County and Zeku County mostly came from the RGLGP’s window (PT_P_ was greater than 40% in both counties). The RGLGP’s restoration measures had outstanding effects in these counties, which has been reported in several case studies of ground-based observations across the project sites (Duojie et al., 2008; Shao et al., 2016a; Xu et al., 2017). However, because the above-mentioned ecological projects only covered a part of the degraded grasslands, the total amount and extent of vegetation restoration in the whole region was relatively limited in analysis using remote sensing (Wang and Rong, 2012). The ECRP, which integrated multiple restoration measures, increased PT_A_ of 70% (12 out of 17) counties in the short-term window compared with the RGLGP. The uptrend took place mostly in the northeast and central parts (**Figure 7D**). Maduo County in the headwater area of the Yellow River showed the most obvious increase (of 9.81%), and the ECRP’s window contributed to 77.24% of the positive turns. This was consistent with the results of studies on the long-term impact of the ECRP (Liangxia et al., 2014; Shao et al., 2017). The possible reason is that the diversified project measures of the ECRP effectively promoted the restoration of degraded alpine grassland in this area (Wang et al., 2020).

It is noteworthy that although the eastern and central parts of the TRHR recovered significantly, some of the southwest region were still in an ecologically fragile state. The PT_A_ of Nangqian, Tanggula and Zaduo County in the three projects’ windows (TNR, RGLGP and ECRP) was low, with the average PT_A_ less than 3.50%. Relevant studies found that the vegetation in these three counties declined during 2003–2005 (Zhai et al., 2020). This could possibly be attributed to the fact that the environment of these counties in the southwestern part was relatively fragile and sensitive (Li et al., 2022); persistent ecological degradation, grassland degradation, and desertification were still present in some places (Liu et al., 2013; Cao et al., 2020; Wang et al., 2023); and the intervention restoration measures were insufficient for this special situation (Wang et al., 2021). Although the ECRP had outstanding performance in our analysis, there were still shortcomings with regard to the grazing prohibition and ecological migration of the ECRP (Du, 2012). The measures implemented in some parts with special natural conditions were unsuitable and unsustainable. Overall, the area showed considerable restoration outcomes under human project intervention. However, we suggest that, it is important to strengthen ecological monitoring and assessment of sensitive and vulnerable areas on the Qinghai–Tibet Plateau in the future. And it is necessary to select effective restoration measures through rapid comparison, detection and identification of immediate or short-term effects, and to expand the area covered by these measures (Shao et al., 2016c). Therefore, our study, which used short-term effects to assess the implementation outcomes and the different responses of various vegetation types to ecological projects, will provide a useful reference for restoring the damaged ecosystem on the Qinghai–Tibet Plateau more efficient through regional dynamic adjustment.

## 5 Conclusions

The study revealed that among the four ecological conservation and restoration projects implemented in the TRHR during 1988–2012, no significant recovery of regional vegetation occurred in a short term following the YRCP. The vegetation gradually recovered from west to east after the implementation of the TNR and the RGLGP. In the ECRP’s window, and a significant short-term effect emerged, because the effect of regional vegetation restoration gradually spread from east to west. There were differences in response of different grassland types to the implemented projects. The restoration of alpine meadow and alpine steppe benefitted more from the RGLGP and the ECRP (all>40%); and the restoration of montane meadow benefitted most from the RGLGP, which represented 47.53%. The ECRP was the most effective for restoring different grassland types and the improvement was relatively balanced (all>46%). Approximately 74.92% of the temperate steppe area improved significantly due to the ECRP. For different counties, the TNR and the RGLGP mainly promoted vegetation restoration in the eastern counties, while the ECRP mainly promoted vegetation restoration in the northeast and central counties. The contribution to vegetation restoration in the counties at the source of the Yellow River was mainly came from the implementation of the ECRP, and 77.24% of the positive turns in Maduo County occurred after that. However, the positive turns in Nangqian, Tanggula and Zaduo County remained low (<3%) after each project, and the regional vegetation in these counties was still needed further intervention and restoration. We believe that evaluating ecological projects requires more attention to identifying and evaluating the short-term effects. Compared with long-term project effects evaluated after a long period of implementation, feedback on short-term effects can guide the dynamic adjustment and continuous improvement of ecological projects through timely feedback.

## Data availability statement

The original contributions presented in the study are included in the article/supplementary material. Further inquiries can be directed to the corresponding author.

## Author contributions

YL provided the idea for this research and contributed to the analysis design. YZ prepared the primary version of manuscript. YL and YZ collected the data, contributed to the data analysis and figure-making. YL contributed to revision for the manuscript. Both authors contributed to the article and approved the submitted version.
